# Analysis of the Role of TpUB05 Antigen from *Theileria parva* in Immune Responses to Malaria in Humans Compared to Its Homologue in *Plasmodium falciparum* the UB05 Antigen

**DOI:** 10.3390/pathogens9040271

**Published:** 2020-04-08

**Authors:** Jerome Nyhalah Dinga, Stephanie Numenyi Perimbie, Stanley Dobgima Gamua, Francis N. G. Chuma, Dieudonné Lemuh Njimoh, Appolinaire Djikeng, Roger Pelle, Vincent P. K. Titanji

**Affiliations:** 1 Biotechnology Unit, Faculty of Science, University of Buea, P O. Box 63 Buea, Cameroon; 2 Department of Biochemistry and Molecular Biology, Faculty of Science, University of Buea, P. O. Box 63 Buea, Cameroon; 3 Biosciences Eastern and Central Africa—International Livestock Research Institute (BecA-ILRI) Hub, P. O. Box 30709 Nairobi, Kenya; 4 Centre for Tropical Livestock Genetics and Health, Royal (Dick) School of Veterinary Studies, The University of Edinburgh, Midlothian, Easter Bush Campus, EH25 9RG Edinburgh, UK; 5 Faculty of Science, Engineering and Technology, Cameroon Christian University Institute, P.O. Box 5 Bali, Cameroon

**Keywords:** Malaria vaccine development, UB05, TpUB05, marker of protective immunity, homologues, cross-species protective immunity

## Abstract

Despite the amount of resources deployed and the technological advancements in molecular biology, vaccinology, immunology, genetics, and biotechnology, there are still no effective vaccines against malaria. Immunity to malaria is usually seen to be species- and/or strain-specific. However, there is a growing body of evidence suggesting the possibility of the existence of cross-strain, cross-species, and cross-genus immune responses in apicomplexans. The principle of gene conservation indicates that homologues play a similar role in closely related organisms. The homologue of UB05 in *Theileria parva* is TpUB05 (XP_763711.1), which has been tested and shown to be associated with protective immunity in East Coast fever. In a bid to identify potent markers of protective immunity to aid malaria vaccine development, TpUB05 was tested in malaria caused by *Plasmodium falciparum*. It was observed that TpUB05 was better at detecting antigen-specific antibodies in plasma compared to UB05 when tested by ELISA. The total IgG raised against TpUB05 was able to block parasitic growth in vitro more effectively than that raised against UB05. However, there was no significant difference between the two study antigens in recalling peripheral blood mononuclear cell (PBMC) memory through IFN-γ production. This study suggests, for the first time, that TpUB05 from *T. parva* cross-reacts with UB05 from *P. falciparum* and is a marker of protective immunity in malaria. Hence, TpUB05 should be considered for possible development as a potential subunit vaccine candidate against malaria.

## 1. Introduction

The malaria parasite has a complex life cycle, consisting of developmental stages in the liver (sporozoites) and blood (merozoites, trophozoites, and schizonts) of vertebrates and Anopheles mosquitos (male and female gametocytes), which serve as transmission vectors [1]. Antigens in the blood-stage of malaria parasites represent targets of parasitic growth and replication, which provoke immune responses that either exacerbate or prevent the growth and development of the parasites in the vertebrate host, hence affecting disease outcomes [2].

Clinical manifestations of malaria are attributed to the blood-stage of parasites that reside within red blood cells (RBCs); thus, vaccines developed against erythrocytic forms of the parasite may contribute considerably to the efficient control of the disease. There are now a number of blood-stage antigens that are being characterized for inclusion as vaccine components in clinical development [3]. Amongst the most studied and advanced blood-stage vaccine antigens, these include circumsporozoite protein (CSP), serine repeat antigen 5 (SERA-5), merozoite surface protein 3 (MSP-3), and apical membrane antigen 1 (AMA1) [3]. These vaccine candidates have not been efficacious in African children [4,5]. However, a multistage vaccine made up of CSP and AMA1 has reduced the incidence of clinical malaria episodes in vaccinated children by 50% compared to a control group [6]. RTS,S (Mosquirix™), a leading vaccine candidate that targets the initial infection of the liver, has only demonstrated a partial efficacy that wanes rapidly [7]. Although a number of erythrocyte-stage antigens are under development, there is still a need to search for better markers of protective immunity and hence, subunit vaccine candidates to improve vaccination outcomes. Second-generation vaccine candidates that could constitute a multivalent rather than univalent vaccine candidate are required, where they could target red-blood-cell-stages and other stages of the parasite [8]. There is, therefore, a need to search other vaccine candidates for the development of a successful malaria vaccine that can confer both full and long-lasting protection.

One approach of identifying better markers of protective immunity would be to seek homologues of markers of protective immunity in closely related organisms. The idea of the conservation of gene products has been proven to suggest that orthologous gene products appear to perform similar functions in closely related species [9]. For example, a circumsporozoite protein (CSP)-based vaccine (VMP001) from *Plasmodium vivax*, containing conserved CSP regions, generated antibodies that could also recognize CSP on the surface of *P. berghei* and *P. falciparum.* Antibodies provoked in mice by recombinant PfCelTOS induced cross-species protection against *P. berghei* challenge by cross-reactivity to heterologous *P. berghei* sporozoites [10]. With the knowledge that homologues may play similar roles in closely related organisms, the information obtained by studying markers of protective immunity could be transferred to another antigen or disease. Conceivably, the homologue of UB05 in *Theileria parva*, TpUB05, has also been shown to be a marker of protective immunity in East Coast Fever (ECF) that affects cattle [11].

In previous studies from our group, the *P. falciparum* antigens UB05, UB09, and chimeric UB05-09 and their respective polyclonal antiserums were analyzed using ELISA, ELISpot assays, and growth inhibition assays with samples from a malaria endemic region. These findings have been published in [12,13,14]. During these same studies, we included TpUB05 and its polyclonal antiserum in separate wells on the same plates and exposed them to the same experimental conditions as the UB05 antigen. In this paper, we present the obtained data and compare the performance of the UB05 antigen from *P. falciparum* as a control antigen to that of TpUB05 from *T. parva* in human malaria.

## 2. Results

### 2.1. Molecular Cloning and Characterization of TpUB05

Using gene-specific primers, *TpUB05* was amplified from *T. parva* schizont RNA using reverse transcription to produce a 291 bp DNA fragment [11], while *UB05* was amplified from a *P. falciparum* cDNA library to produce a 243 bp DNA fragment [12]. Both fragments were cloned into a pET32a+ expression vector and overexpressed in *Escherichia coli* cells, yielding recombinant fusion proteins migrating at 28 KDa and 26 KDa, respectively, in SDS-PAGE performed in 15% polyacrylamide. The recombinant fusion proteins contained a 6xHis tag, a S tag domain, and a 109 amino acid thioredoxin fusion protein partner. However, it was easier to overexpress and purify TpUB05 as compared to UB05.

### 2.2. TpUB05 Antigen from T. parva Possesses B-Cell Epitopes that Cross-Reacted with Human Antibodies in a Malaria Endemic Area

**Figure 1:**
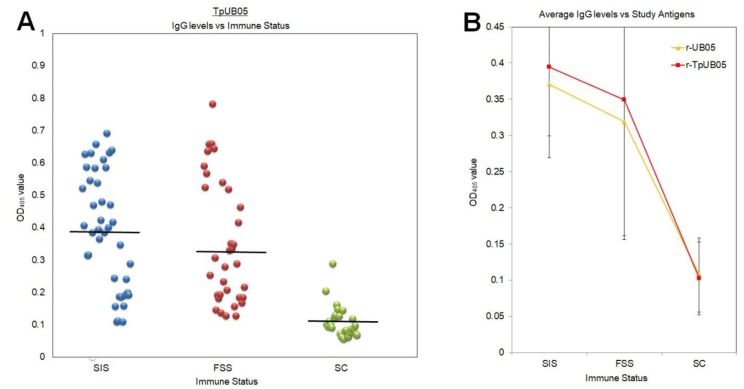
Antibody response of TpUB05 in humans. (**A**) Comparison of antigen-specific antibodies to TpUB05 in plasma collected from human subjects (semi-immune status (SIS), frequently sick status (FSS), and sick children (SC)). Plasma from SIS (40 subjects) contained more TpUB05-specific antibodies compared to FSS and SC (61 subjects; *p* = 0.0001 using analysis of variance (ANOVA)). Bars represent the mean optical density (OD)_405_ value. (**B**) Average OD_405_ values of TpUB05 and UB05 were observed with the different immune status groups. The data show that there is no biological difference between anti-TpUB05 and anti-UB05 antibodies levels even though the *p*-value indicates otherwise. Error bars represent standard deviation.

Forty (40) semi-immune status (SIS), 34 frequently sick status (FSS), and 27 sick children (SC) samples were obtained; thus 101 samples were analyzed using ELISA. Plasma from the study subjects was tested with TpUB05 using ELISA and it was shown that SIS possessed greater amounts of antibodies compared to FSS. However, the difference was not observed to be statistically significant using analysis of variance (ANOVA) and Friedman tests. Adults (SIS + FSS) produced (average optical density (OD) value of 0.372) 3.62 times more antigen-specific antibodies to TpUB05 (*p* = 0.0001) than children (SC; average OD value of 0.102; Figure 1A).

There was a statistically significant difference between TpUB05 and UB05 for all 101 subjects (SIS, FSS, and SC; paired samples *t*-test) and for the 74 adults (SIS and FSS; Wilcoxon signed rank test). Both tests gave a *p*-value of 0.006 (Figure 1A,B).

However, looking at the average OD values obtained for SIS (0.39 for TpUB05 and 0.37 for UB05) and FSS (0.34 for TpUB05 and 0.31 for UB05), the difference, which is statistically significant, does not appear to be biologically significant; however, the data suggest cross-reaction (Figure 1B).

### 2.3. Human Antibody Levels to TpUB05 Negatively Correlate with Fever and Malaria Parasitemia

**Figure 2:**
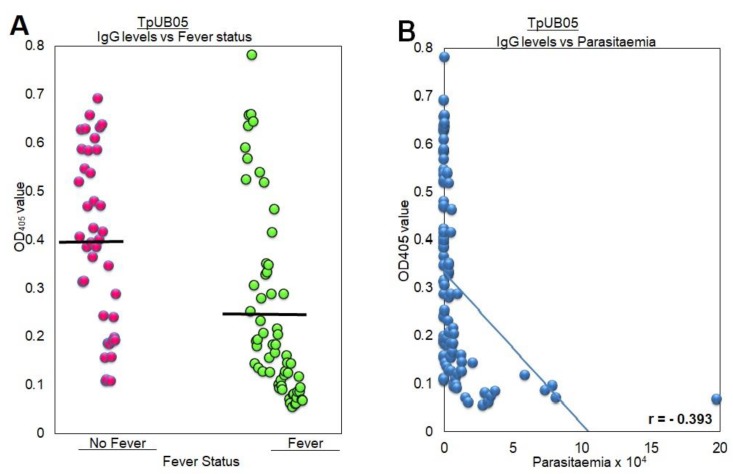
Relation between fever, parasitemia, and antibody response to TpUB05. (**A**) Comparing the absence or presence of fever with the anti-TpUB05 antibody level in human plasma (*p* = 0.0001). Subjects without fever had significantly higher levels of antigen-specific antibodies than those with fever. This significant difference was determined using ANOVA. (**B**) The relationship between anti-TpUB05 antibody levels and parasite load indicates there is a negative correlation (*r* = −0.393, *p* = 0.001). Pearson correlation was used to analyze the correlation.

When the antibody levels to TpUB05 were plotted as a function of parasitemia, it was observed that that there was a negative correlation between parasitemia and antibody levels. This implies that the higher the specific antibody level, the lower the parasite density in the blood. In other experiments, antibody levels have been shown to be statistically higher in subjects without a fever than in subjects with a fever (Figure 2A,B). With *r* = −0.393 and a significant *p*-value of 0.0001, there is a significant negative correlation between TpUB05-specific antibody levels and parasite load. What is driving this result is the one subject presenting 20 × 10^4^ parasitemia and the four subjects presenting around 5–10 × 10^4^ parasitemia. If those subjects are removed from the analyses, the correlation would disappear. Taken together, these results suggest that the responses to the two antigens are very similar.

### 2.4. Tpub05 Stimulates T-Cell Responses of Semi-Immune Malaria Patients Greater than Those of Malaria Susceptible Patients

**Figure 3:**
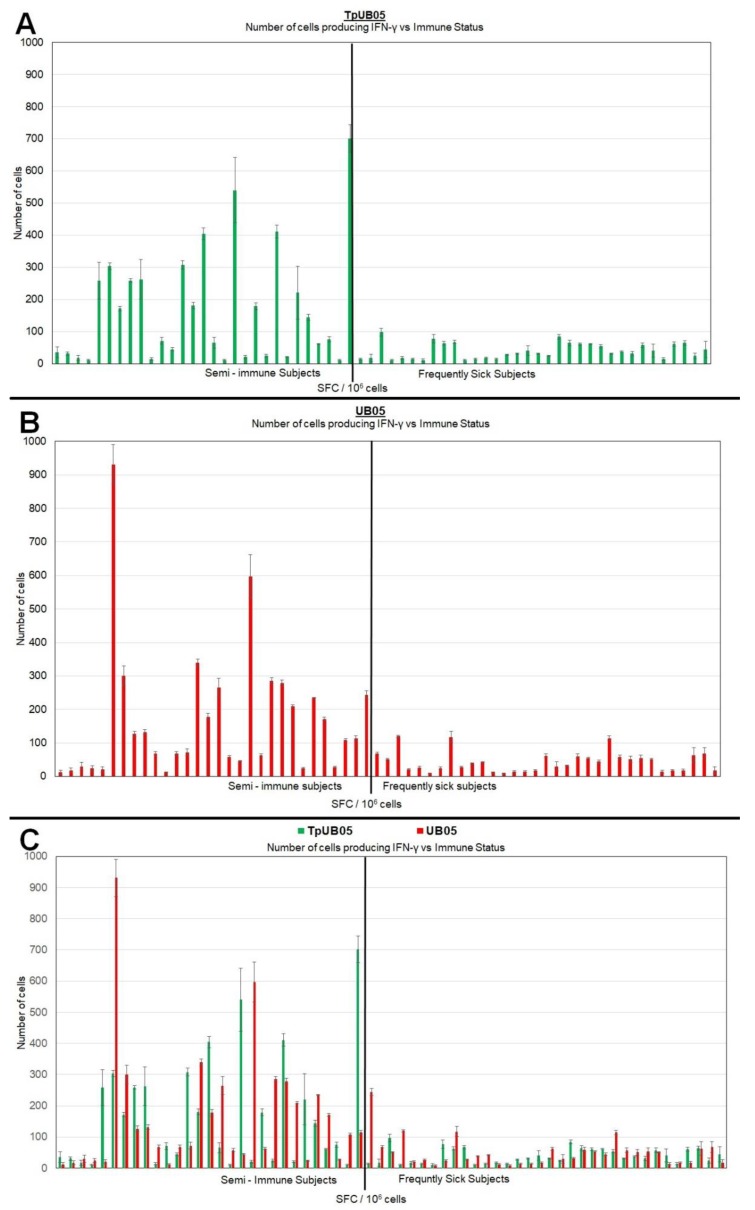
Human ELISpot assay using TpUB05 to stimulate human peripheral blood mononuclear cells (PBMCs) for IFN-gamma production. Spot-forming cells per million (SFC/10^6^ cells) was used to assess the magnitude of response. (**A**) r-TpUB05 from *T. parva* was used to stimulate human T-cells from 63 subjects (35 SIS and 28 FSS) with SIS having more SFC than FSS (*p* = 0.0001 using *t*-test). (**B**) Human ELISpot assay using r-UB05 from *P. falciparum*. (**C**) Combined SFC/10^6^ cells data from both TpUB05 and UB05 antigens. Comparison showed there was no difference in the magnitude of response provoked by both study antigens. Error bars represent standard deviations.

In an attempt to investigate the possible ability of TpUB05 in T-cell mediated immunity we compared the T-cell responses of cohorts of semi-immune and susceptible malaria patients to these orthologous antigens. Sixty-three (63; consisting of 35 SIS and 28 FSS) peripheral blood mononuclear cell (PBMC) samples were used for a human ELISpot assay. More PBMCs from SIS produced IFN-γ than FSS when stimulated with the TpUB05 antigen (*p* = 0.0001 using *t*-test; Figure 3A). When the recognition of TpUB05 was compared to that of UB05 (Figure 3B), there was no significant difference (Figure 3C).

The determination of spot-forming cells per million (SFC/10^6^ cells) was also used to assess the difference between TpUB05 and UB05 in recalling T-cell function by IFN-γ production. The magnitude of the response showed TpUB05 provokes more cells from SIS (average of 168 SFC/10^6^ cells) to produce IFN-γ than FSS (average of 40 SFC/10^6^ cells). The same trend was observed for UB05 (average of 166 SFC/10^6^ cells for SIS and 49 SFC/10^6^ cells for FSS; Figure 3A–C). The positive controls had values ranging from 6993 to 8476 SFC/10^6^ cells (data not shown).

**Figure 4:**
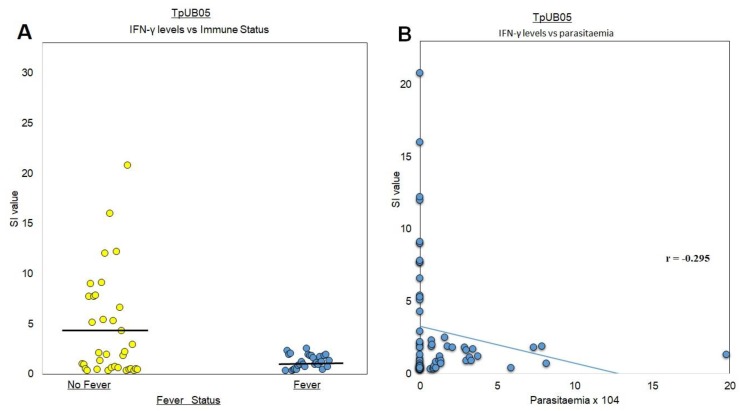
Relationship between T-cell responses (IFN-γ production) to recombinant TpUB05, fever, and parasitemia. (**A**) Subjects with no fever (SIS) appear to produce more IFN-gamma in response to stimulation by TpUB05 compared to those with a fever (FSS; *p* = 0.002 using ANOVA). Bars represent mean values. (**B**) Production of IFN-gamma is associated with a protective immune response against malaria amongst these adults. Pearson correlation analysis showed *r* = −0.295, *p* = 0.01.

Using stimulatory index (SI), we also found significantly higher IFN-γ production by PBMCs from subjects with no fever (*p* = 0.002) compared to those who had a fever (Figure 4A). The same trend was observed when the SI values were compared to the absence or presence of parasitemia. (Figure 4B). These results suggest that TpUB05 is a marker for protective immunity to malaria due to preferential stimulation of the production of IFN-γ in SIS.

### 2.5. Rabbit Polyclonal Antibodies Raised against Recombinant TpUB05 Perform Better in Inhibiting P. falciparum Parasite Growth in Vitro than Antibodies to Recombinant UB05

**Figure 5:**
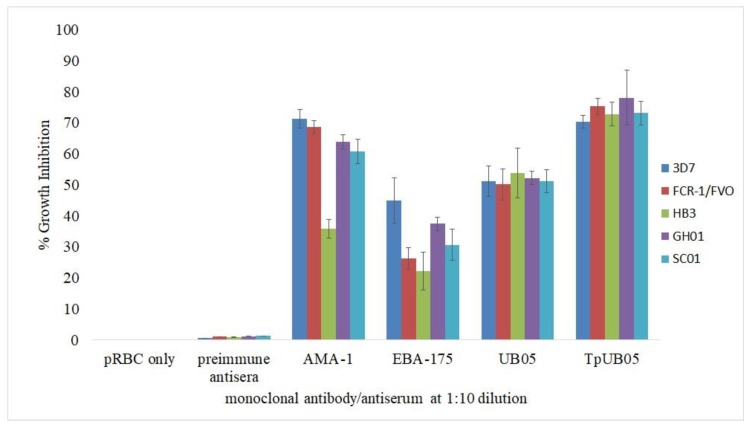
Comparing anti-TpUB05 and anti-UB05 polyclonal antiserum in a growth-inhibition assay. Rabbit antisera against rTpUB05 and rUB05 were used in vitro to test for their ability to inhibit parasite growth. The anti-TpUB05 antiserum performed better than anti-UB05 antiserum in inhibiting in vitro parasite growth (*p* = 0.0001 using ANOVA). This was done using *P. falciparum* laboratory strains, 3D7, FCR-1/FV0, and HB3, and two field isolates, GH01 and SC01. They were tested at a 1:10 dilution. The experiment was run in triplicate and repeated once. Error bars represent standard deviations.

The ability of the TpUB05-specific antiserum to inhibit parasite growth in vitro was tested using a growth-inhibition assay (GIA), as described in the Materials and Methods section, and the data were compared with those obtained with the UB05-specific antiserum. Rabbit anti-TpUB05 antiserum was able to inhibit parasite growth in vitro significantly better than the performance of the anti-UB05 antiserum (*p* = 0.0001 using ANOVA; Figure 5). This inhibition was observed for all the parasite strains tested (Figure 5).

In the positive control experiments, we tested in parallel two monoclonal antibodies available from BEI Resources, namely, anti-AMA1 and anti-EBAI1. Amongst the parasite strains tested, only the *P. falciparum* HB3 strain was not inhibited by the positive control monoclonal antibodies (anti-AMA1 and anti-EBA175), indicating that these two are less cross-reactive than those directed against TpUB05 and UB05. The results presented in Figure 5 show that anti-TpUB05 antiserum does not inhibit parasite growth significantly more than anti-AMA1 antibody. However, it performed better than the anti-EBA175 antibody in inhibiting in vitro parasite growth (*p* = 0.0001 using ANOVA).

### 2.6. Online Biometric Prediction Indicates the Presence of TpUB05 Epitopes Capable of Binding to Human Major Histocompatibility Complex (MHC) Epitopes

The online prediction of human T-cell and antibody epitopes revealed the presence of epitopes that could bind to human MHC on TpUB05. MHC I and II binding peptides with a percentile rank below 1.0 were considered as those with a very high affinity for the MHC molecules (Supplementary Table S1). The lower the percentile rank, the stronger the binding affinity. Using algorithms from www.iedb.org, we also predicted the presence of human antibody epitopes in TpUB05 (Supplementary Table S1).

## 3. Discussion

Malaria subunit vaccine development has mostly focused on single antigens. However, the complex nature of the malaria parasite life cycle and the mechanisms it uses to evade human immune response implies the ideal malaria vaccine should target several antigens expressed at different stages of the parasite’s development [8], as well as homologous antigens from related species. Such a vaccine would circumvent the setbacks observed with strain-specific or species-specific protective immune responses. The first step towards this would be the identification and characterization of homologous antigens from phylogenetically related organisms as markers of protective immunity against malaria. Here, we have shown for the first time that the TpUB05 antigen from *T. parva* cross-reacts with UB05 and is associated with protective immunity in malaria.

The UB05 antigen has previously been compared to its chimeric construct, UB05-09, using an ELISpot assay, ELISA, and growth-inhibition assay, published in [12,13,14,15], respectively. During those experiments, TpUB05 was added into separate wells on the same plates and exposed to the same experimental conditions as the other study antigens/antisera. However, the results of these experiments on TpUB05 are only reported in the present paper. Since the data on UB05 have previously been published, we are now using them as control data to compare the provoked and detected immune responses to those provoked or detected by its homologue in *T. parva*, TpUB05. From all samples from which PBMCs were isolated, plasma was also collected for ELISA, but not all ELISA samples were collected from samples from which PBMCs were isolated. Hence, more samples were analyzed using ELISA than ELISpot assays.

The principle of the conservation of gene functions shows that most orthologous gene products play a similar role in closely related organisms [9]. We decided to characterize TpUB05 in malaria caused by *P. falciparum.* This observation of effective cross-immunization with homologous antigens has been shown previously [9]. In other words, it might be more useful to search for antigens that induce protection against ECF and test malaria caused by a distant relative, such as *P. falciparum*. To verify this hypothesis, we carried out the simultaneous testing of immune responses in semi-immune and malaria-susceptible subjects and found that the TpUB05 antigen from *T. parva* cross-reacts with the UB05 antigen from *P. falciparum*.

Although B-cells have been seen to contribute little to resistance and protective immunity for infections with apicomplexans, many studies have shown that hosts infected with these parasites are capable of producing parasite-specific immunoglobulins which are protective after recovering from an infection [16]. The present study shows that antibodies in human plasma recognized TpUB05. The significant *p*-values obtained when antibody levels/magnitude of IFN-γ production were compared to fever status, as well as the significant negative correlation observed between antibody levels/magnitude of IFN-γ production and parasitemia, indicate that immune responses to TpUB05 are associated with protection against malaria caused by *P. falciparum*.

Studies to elucidate the mechanism(s) of the protective immune response to apicomplexan parasites implicate the role of the production of gamma interferon (IFN-*γ*), amongst other cytokines and chemokines [17], which control parasite infectivity and interfere with parasite development. Biostatistical analyses have revealed that there are T-cell epitopes in TpUB05 that could bind and be recognized by human T-cells, leading to the production of IFN-gamma. This production of IFN-gamma occurs in a manner that is associated to immune protection against malaria, as it is preferentially recognized by semi-immune subjects compared to frequently sick subjects. The significant *p*-values obtained when magnitudes of IFN-γ production were compared to fever status, as well as the significant negative correlation observed between magnitude of IFN-γ production and parasitemia, indicate that immune responses to TpUB05 are associated with protection against malaria. When these data were compared to those obtained with the UB05 antigen from *P. falciparum* [12], which were obtained under the same experimental conditions, the same trend of correlation with protection against malaria was observed. There was no significant difference between these two antigens in stimulating the production of IFN-gamma from human PBMCs, suggesting that they could stimulate similar reactions in vivo; however, this remains to be shown.

The hallmark of an effective malaria subunit vaccine would be its ability to stimulate the cellular and antibody components of the immune system that are protective. This implies that an antigen’s ability to preferentially detect antigen-specific antibodies and recall T-cell function in people who have acquired limited protective immunity to malaria is an indication that the protein is involved in immune protection against malaria parasites. TpUB05 was able to recall T-cell ability to produce IFN-gamma in SIS subjects and detect antigen-specific antibodies in these subjects, hence, it is associated with protective immunity in malaria.

A normal body temperature with little or no parasites in the blood, as well as higher IFN-gamma production and antibody amounts, have been shown to correlate with immune protection against clinical malaria [18,19,20]. The data obtained in this study strongly suggest that TpUB05 is associated with protective immunity in clinical malaria.

The contamination of antigen preparations with lipopolysaccharide (LPS) has been shown to confound T-cell response assay results to malaria antigens. However, it is unlikely that there was a contribution of possible contaminating bacterial LPS to the observed responses in the present study, as the responses were specific to each of the groups and as the control antigen (UB05) was prepared under similar conditions and treated in the same way as the test antigen (TpUB05). Cytokines whose production appear to be influenced by LPS include IL-1beta and IL-6. However, IFN-gamma, whose production is not affected by LPS, was studied herein [21].

The present study shows that TpUB05 possesses T-cell and B-cell epitopes that bind and recognize human MHC molecules and antibodies, respectively. This was confirmed using in silico algorithms. It was then necessary to determine if the polyclonal antibodies raised against TpUB05 from *T. parva* have any effect on malaria parasite development by employing an in vitro growth-inhibition assay. The growth-inhibition assay, which involves impaired merozoite invasion and the subsequent development of parasites in erythrocytes, is currently being considered as one of most relevant assays to screen potential blood-stage vaccine candidates prior to moving to the stage of clinical development. Purified polyclonal total IgG induced in rabbits against TpUB05 from *T. parva* was able to significantly inhibit malaria parasite growth in vitro. This inhibition was statistically higher than that observed with the anti-UB05 polyclonal antibody as well as the anti-EBA175 monoclonal antibody, but not the anti-AMA1 monoclonal antibody. Hence, TpUB05 should be considered to be associated with protective immunity against malaria.

A bioinformatics comparison of TpUB05 and UB05 shows that they exhibit a degree of sequence homology, where 43.3% identity and 67% similarity was observed. The in silico analysis predicted the presence of human T-cell and antibody epitopes of TpUB05 and the cross-reactivity observed was expected, in view of the significant homology between the two antigens. However, TpUB05 may contain more potent B-cell epitopes compared to UB05 that are yet to be identified.

Altogether, the results from this study imply that TpUB05 is associated with protection against malaria, and this confirms the notion that homologues could play similar roles in related organisms. This is in line with previous studies where *Mycobacterium bovis* (BCG vaccine) was used to vaccinate humans against *M. tuberculosis* [22] and where humans exposed to *Onchocerca ochengi* were protected against *O. volvulus* infection [23]. The potential role of TpUB05 in inducing an effective immune protection against malaria infection and disease warrants more investigation.

The importance of immune cross-reactivity may indicate the enhancement of protective immune responses amongst distantly related apicomplexans such as *P. falciparum* and *T. parva*. There is a strong cross-reactivity between TpUB05 and UB05 antigens, based on ELISA, ELISpot, and growth-inhibition data, strongly suggesting the use of TpUB05 for the development of a malaria vaccine.

## 4. Materials and Methods

### 4.1. Study Site and Design

The study was carried out in Buea, which is an endemic center for malaria. Buea is a multi-ethnic town found along the flanks of Mount Cameroon in the southwestern region of the Republic of Cameroon. The prevalence of malaria in Buea varies between meso-endemic (dry season) and hyperendemic (rainy season) zones with perennial malaria transmission [24].

The UB05 antigen has previously been tested and compared to its chimeric construct, UB05-09 and published [12,14,15]. During those experiments, the TpUB05 antigen was added into separate wells on the same plates and exposed to the same experimental conditions as the other study antigens/antisera. However, the data obtained with TpUB05 are only reported in the present paper. We are now using the data on UB05 as control data since it has already been published. So the immune responses detected by UB05 is being compared to those provoked by its homologue in *T. parva*: TpUB05 (Table 1).

### 4.2. Study Population

The following criteria were used to screen and recruit subjects into the study, as earlier published: (i) Subjects who were aged 18 years of age or older and who had been living in the study site for at least 3 years; (ii) subjects with no history of a malaria episode in the last 12 months, no fever, and parasitemia at sample collection, no use of a mosquito bed net, hence being exposed to mosquito bites, and no prophylaxis were designated to semi-immune subjects (SIS); (iii) subjects who had had at least one malaria episode in the last 12 months and had a fever an parasitemia at the time of sample collection were referred to as frequently sick subjects (FSS); and (iv) children aged five or younger who had a fever and parasitemia at the time of sample collection were referred to as sick children (SC). This cohort of individuals was highly selective and has been described previously [12,16]. The analyses for fever, parasitemia, and blood sample collection for ELISpot assay and ELISA took place between March and June of 2014 [12].

### 4.3. Assessment of Nutritional Status

It has been shown that being in good health reduces susceptibility to non-communicable and infectious diseases, including malaria [25]. The assessment of the nutritional status of the study population was done, as it is assumed that a probable indication of good health and being healthy is having a normal body mass index (BMI) [26]. Only subjects in the normal or pre-obese range were admitted into the study [12,27]. Underweight or obese persons were excluded in the study. BMI was calculated by the following formula: BMI (kg/m^2^) = weight (kg)/height^2^ (m)

### 4.4. Parasite Strains, Antigens, and Polyclonal Antibody

As previously mentioned [15], all laboratory strains were generously donated by the MR4/BEI Resources, NIAID, NIH, Manassas, VA. The following *P. falciparum* laboratory strains were used: FCR-1/FVO (MRA-909, contributed by W. Trager), 3D7 (MRA-102, contributed by Daniel J. Carucci), and HB3 (MRA-155, contributed by Thomas E. Wellems) for the in vitro assay. Two field isolates, GH01 and SC01, were also used for the study, and were obtained from the Buea District Hospital and Solidarity Clinic, Buea, Cameroon, respectively. The overexpression of recombinant TpUB05 and its polyclonal antibody production in rabbits has been previously described [11].

### 4.5. Preparation of Peripheral Blood Mononuclear Cells from Human Donors

The procedure to isolate peripheral blood mononuclear cells (PBMCs) was performed according to manufacturer’s instructions. Briefly, the preparation of PBMCs for use in the ELISpot assay was done using the Percoll gradient method, as earlier described [28]. Briefly, 8 mL of venous blood was collected from subjects in EDTA-containing tubes. An equal volume of sterile PBS was then added to the blood samples and layered on a Percoll discontinuous gradient solution (5 mL each of 60%, 50%, and 40% Percoll solution, layered in that order). The samples were centrifuged at 11,709 × g for 30 min. The white buffy coats at the 40%/50% and the 50%/60% interfaces were carefully collected and put into a 50 mL falcon tube. These interfaces contain monocytes and lymphocytes, respectively. Washing of the collected buffy coats to remove excess Percoll was done twice with 40 mL of sterile PBS supplemented with 5% fetal calf serum at 4000 rpm for 10 min. Cells were then re-suspended in 2 mL of complete R.P.MI-1640 culture medium (CCM), and cell viability and quantification was done using Trypan blue staining and an improved Neubauer counting chamber [29].

### 4.6. Enzyme-Linked Immunosorbent Spot (ELISpot) Assay

The enzyme-linked immunosorbent spot assay was carried out as described [12]. Briefly, the ELISpot assay was used to determine the proportion of IFN-gamma-secreting PBMCs from subjects ex vivo upon stimulation with recombinant antigens TpUB05 and UB05. The PBMCs were used within 2 h of collection in EDTA-containing tubes, that is, they were transported to the laboratory (10 min away), isolated, and analyzed. Cell and antigen preparations were carried out in a biological safety cabinet prior to use to ensure the sterility of the cells and protein samples. Here, 5 µg/mL of each antigen was mixed with 300,000 PBMCs and tested in triplicate. MABTECH AB kits were used to carry out the ELISpot assay, as previously described [27]. The positive control used to recall T-cell memory was the anti-CD3-2 monoclonal antibody. Negative controls were wells with PBMCs and PBS only (no antigen). The stimulation index (SI) was calculated as follows: SI = mean number of spots in triplicate test (with antigen) wells divided by mean number of spots in triplicate negative control (without antigen) wells. An SI value of more than two was considered positive [30,31]. SFC/10^6^ cells were also used to determine the magnitude of response by PBMCs. The antigen-specific spots count was calculated as the mean of three wells minus the mean spot count from PBS negative control wells. Spot counts were multiplied by 3.33 to obtain spot-forming cells per million cultured PBMCs. All analyses were performed with these final counts, i.e., spots forming cells per million cultured PBMCs.

### 4.7. Plasma Sample Collection

Plasma was collected from the blood samples and stored until further analysis. At the time of performing the ELISA experiments, additional subjects were recruited into the study to obtain more plasma samples and increase the sample size for analysis. Plasma samples were also collected in EDTA tubes from children below the age of five whose parents gave their consent and tested positive for malaria (fever and parasitemia). The samples were then centrifuged and the plasma was aspirated and stored at −20 °C until use.

### 4.8. Determination of the Levels of Antibodies Recognizing r-TpUB05 and r-UB05 By ELISA

Antibody (total IgG) measurement was carried out by ELISA, as earlier described [32], with some modifications. Here, we used 100 µL of 0.625 µg/mL r-TpUB05 or r-UB05 in PBS, which was used to coat microtiter plates by incubation at 4 °C overnight. Some wells were coated with a soluble fraction of crude *E. coli* extract or the r-UB05 antigen as a positive control for the antigen, while the tag-only (Fusion partner) antigen was used as a negative control antigen. The plates were washed thrice with 200 μL of a wash buffer (PBS-Tween-20, 0.05%) after overnight incubation, followed by blocking with 150 μL per well of 0.2% casein in PBS with 0.05% Tween-20. After the second washing, plasma was added at 1:150 dilutions in the wash buffer, containing 1% skimmed milk. The samples were then incubated at room temperature for 3 h. To obtain and record background signals (negative control for plasma), human plasma from malaria-naïve individuals, which was kindly given to us by Mrs. Philomena Gwanmesia of the Biotechnology Center, University of Yaounde I, Cameroon, was included in the assay. After the 3 h incubation at room temperature, the plates were washed with the wash buffer three times and 100 μL of the anti-rabbit IgG-HR.P. conjugate was diluted at 1:10,000 in a wash buffer containing 1% skimmed milk and then incubated for 1 h at room temperature, after which the plates were washed with the wash buffer and 100 μL of the substrate added. The optical densities (OD) of the wells were obtained at 405 nm using a microplate reader. A subject was considered positive if its OD value was equal to or greater than the mean control OD + 2 Standard Deviation (2SD). The experiment was run in duplicate.

### 4.9. Growth Inhibition Assay

The ability of purified rabbit IgGs induced against r-TpUB05 and r-UB05 to inhibit the replication of *P. falciparum* in vitro was tested by measuring parasite lactate dehydrogenase (pLDH) in the late trophozoite- /early schizont-stages in cultures, as described in Methods In Malaria Research 6th Edition [33]. Briefly, the enrichment of late-stage infected erythrocytes was done by performing three 5% sorbitol synchronizations and 60% Percoll gradient centrifugations. The assay was performed using late trophozoite- or schizont-stages. This was ensured by starting and stopping (at approximately 96 h) the experiment when most of the parasites were at those stages. The start parasitemia of the culture was kept at 0.1%–0.3% parasitemia and 1% hematocrit in a CO_2_ incubator for two cycles. Polyclonal antibodies raised against r-TpUB05 or r-UB05 were added at the start of the cultured at an optimized dilution of 1:10 (final concentration of anti-TpUB05 total IgG = 0.48 mg/mL and anti-UB05 total IgG = 0.63 mg/mL). The monoclonal antibodies, anti-EBA-175 RII (final concentrations; R217 at 0.133 mg/mL and R218 at 0.153 mg/mL), and anti-AMA1 (final concentration of 0.1 mg/mL), were used as positive controls in the inhibition assays, while the negative control wells contained pre-immune sera (final concentration of 0.7 mg/mL) or no antiserum. The control wells were included in the assay that contained either only parasitized RBCs (pRBC only) or normal RBCs. Percentage growth inhibition was calculated by subtracting the average OD from the normal RBCs wells from all the other OD values obtained before proceeding to the calculation proper.

### 4.10. Immune Epitope Prediction using Online Resources

The in silico prediction of immune epitopes from TpUB05 and UB05 was done using algorithms from IEDB Analysis Resources (www.immuneepitope.org). The MHC I binding predictions were made on 7/30/2019 using the IEDB recommended method, which combines predictions from ANN [34,35,36,37], SMM [38], and Comblib [39]. The MHC II binding predictions were made on 7/30/2019 using the IEDB analysis resource consensus tool [40]. The Emini surface accessibility scale [41], Kolaskar and Tongaonkar antigenicity scale [42], BepiPred-1.0 linear epitope prediction [43], and BepiPred-2.0 sequential B-cell epitope predictor [44] were used to predict the presence of human antibody epitopes on TpUB05.

### 4.11. Statistical Analysis

The analysis of variance test was employed to compare differences between the mean OD and SI values between study groups. The Kruskal–Wallis test was used to assess differences between the body temperature, parasitemia, OD, and SI values. All these tests were performed using the SPSS software (Version 17.0, Chicago, IL, USA). A value of *p* < 0.05 was considered significant.

### 4.12. Ethics Approval and Consent to Participate

Ethical clearance for this study (Ref.: 2013/144/UB/FHS/IRB) was obtained from the Institutional Review Board of the Faculty of Health Sciences, University of Buea. All participating adult subjects read, approved, and signed the consent form. A parent or guardian of any child participating in the study provided informed consent on the child’s behalf. The informed consent given was written. Ethical clearance for blood collection and polyclonal antibody production in rabbits was approved by the International Livestock Research Institute—Institutional Animal Care and Use Committee (ILRI-IACUC; ref. no. 2013.05). ILRI-IACUC provided clearances based on “The Animal Research: Reporting In Vivo Experiments” (ARRIVE) guidelines on the care and use of animals in research.

## Figures and Tables

**Table 1:**
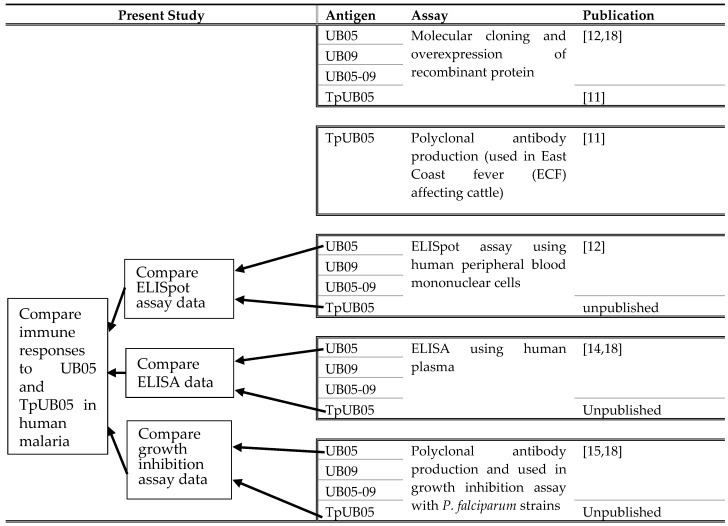
Study design to show how the study was planned, executed, and reported.

## Supplementary Materials

The following are available online at https://www.mdpi.com/2076-0817/9/4/271/s1, Table S1: Predicted human T-cell and B-cell epitopes predicted on TpUB05 antigen Immune epitopes on the TpUB05 antigen were predicted in silico using algorithms found on www.iedb.org. MHC I and II binding peptides presented here are those with a percentile rank below 1.0. Percentile rank below 1.0 were considered as those with a very high affinity for the MHC molecules. The lower the percentile rank the stronger the binding affinity. These would have to be tested in vitro and in vivo to confirm their immunogenicity.
