# Supplementary material for: Analysis of the Role of TpUB05 Antigen from Theileria parva in Immune Responses to Malaria in Humans Compared to Its Homologue in Plasmodium falciparum the UB05 Antigen
Source: Pathogens. 2020 Apr 8;9(4):271. doi: 10.3390/pathogens9040271 (PMC7238281; doi:10.3390/pathogens9040271)
Supplement: Supplementary file 1 [file pathogens-09-00271-s001.pdf]

**<u>Supplementary Table 1</u>:** Predicted human T cell and B cell epitopes predicted on TpUB05 antigen

Immune epitopes on TpUB05 antigen were predicted *in silico* using algorithms found on www.iedb.org. MHC I and II binding peptides presented here are those with a percentile rank below 1.0. Percentile rank below1.0 were considered as those with a very high affinity for the MHC molecules. The lower the percentile rank the stronger the binding affinity. These would have to be tested *in vitro* and *in vivo* to confirm their immunogenicity.

| No. | MHC I binding peptides | MHC II binding peptides | Antibody epitopes |
|---|---|---|---|
| 1 | FEFNLTHSLL | LSCTFLFMFGELLRL | TKRKPHST |
| 2 | FEFNLTHSL | SCTFLFMFGELLRLM | PHSTSFV |
| 3 | KGLNRLFPFR | CTFLFMFGELLRLMN | HSTSFVD |
| 4 | FLFMFGELLR | TFLFMFGELLRLMNN | STSFVDL |
| 5 | STSFVDLTR | FLFMFGELLRLMNNL | TSFVDLT |
| 6 | VLLSCTFLF | LFMFGELLRLMNNLE | SFVDLTR |
| 7 | RLFPFRRNF | FRRNFEFNLTHSLLF | FVDLTRF |
| 8 | FPFRRNFEF | TVLLSCTFLFMFGEL | DLTKRKPHS |
| 9 | GLNRLFPFR | SCTFLFMFGELLRLM | VDLTRFL |
| 10 | HSTSFVDLTR | CTFLFMFGELLRLMN | DLTRFLD |
| 11 | SVCVLLHSFR | TFLFMFGELLRLMNN | LTRFLDS |
| 12 | FMFGELLRLM | FMFGELLRLMNNLEF | TRFLDSG |
| 13 | FMFGELLRL | RRNFEFNLTHSLLFS | RFLDSGV |
| 14 | VLLSCTFLF | LTLFTVLLSCTFLFM | FLDSGVL |
| 15 | SVCVLLHSFR | LSCTFLFMFGELLRL | LDSGVLT |
| 16 | LFPFRRNFEF | CTFLFMFGELLRLMN | SGVLTLFTVLLSCTFL |
| 17 | SVCVLLHSFR | TFLFMFGELLRLMNN | FTVLLSC |
| 18 | FLFMFGELLR | SCTFLFMFGELLRLM | TVLLSCT |
| 19 | FPFRRNFEF | CTFLFMFGELLRLMN | VLLSCTF |
| 20 | FLDSGVLTL | TFLFMFGELLRLMNN | LLSCTFL |
| 21 | FMFGELLRL | VLLSCTFLFMFGELL | LSCTFLF |
| 22 | KGLNRLFPF | LLSCTFLFMFGELLR | SCTFLFM |
| 23 | TVLLSCTFLF | RNFEFNLTHSLLFSV | CTFLFMF |
| 24 | NLTHSLLFSV | SCTFLFMFGELLRLM | TFLFMFG |
| 25 | RNFEFNLTH | FLFMFGELLRLMNNL | FLFMFGE |
| 26 | KPHSTSFVDL | FLFMFGELLRLMNNL | LFMFGEL |
| 27 | FLFMFGELL | LFMFGELLRLMNNLE | FMFGELL |
| 28 | RLMNNLEFL | FMFGELLRLMNNLEF | MFGELLR |
| 29 | LLFSVCVLL | MFGELLRLMNNLEFL | HSLLFSVCVLLHS |
| 30 | LFMFGELLR | FGELLRLMNNLEFLN | FGELLRL |
| 31 | LFPFRRNFEF | GELLRLMNNLEFLNH | GELLRLM |
| 32 | TSFVDLTRF | LSCTFLFMFGELLRL | ELLRLMN |
| 33 | RFLDSGVLTL | NFEFNLTHSLLFSVC | LLRLMNN |
| 34 | CVLLHSFRR | LSCTFLFMFGELLRL | LRLMNNL |
| 35 | FMFGELLRL | CTFLFMFGELLRLMN | RLMNNLE |
| 36 | LTHSLLFSV | ELVKKGLNRLFPFRR | LMNNLEF |
| 37 | FPFRRNFEF | LVKKGLNRLFPFRRN | MNNLEFL |
| 38 | GELLRLMNNL | VKKGLNRLFPFRRNF | NNLEFLN |
| 39 | LEFLNHELV | KKGLNRLFPFRRNFE | MNNLEFLNHELVKK |
| 40 | RLMNNLEFL | KGLNRLFPFRRNFEF | |
| 41 | VLLSCTFLFM | FLFMFGELLRLMNNL | |

| | | | |
|---|---|---|---|
| 42 | CVLLHSFRR | LFMFGELLRLMNNLE | |
| 43 | LNRLFPFRR | TFLFMFGELLRLMNN | |
| 44 | LTHSLLFSV | SCTFLFMFGELLRLM | |
| 45 | LFTVLLSCTF | FEFNLTHSLLFSVCV | |
| 46 | FTVLLSCTF | ELLRLMNNLEFLNHE | |
| 47 | RLMNNLEFL | GLNRLFPFRRNFEFN | |
| 48 | FNLTHSLLF | LNRLFPFRRNFEFNL | |
| 49 | FLDSGVLTLF | FLFMFGELLRLMNNL | |
| 50 | FEFNLTHSL | LFMFGELLRLMNNLE | |
| 51 | FEFNLTHSL | SLLFSVCVLLHSFRR | |
| 52 | FPFRRNFEF | LLFSVCVLLHSFRRS | |
| 53 | STSFVDLTR | LFSVCVLLHSFRRSN | |
| 54 | STSFVDLTRF | GVLTLFTVLLSCTFL | |
| 55 | LLRLMNNLEF | SGVLTLFTVLLSCTF | |
| 56 | TFLFMFGEL | VLTLFTVLLSCTFLF | |
| 57 | NLTHSLLFSV | LTLFTVLLSCTFLFM | |
| 58 | KKGLNRLFPF | TLFTVLLSCTFLFMF | |
| 59 | FLDSGVLTL | LFTVLLSCTFLFMFG | |
| 60 | LLSCTFLFM | FTVLLSCTFLFMFGE | |
| 61 | KGLNRLFPF | LSCTFLFMFGELLRL | |
| 62 | FPFRRNFEFN | EFNLTHSLLFSVCVL | |
| 63 | NLTHSLLFSV | LFMFGELLRLMNNLE | |
| 64 | FLDSGVLTL | STSFVDLTRFLDSGV | |
| 65 | FLFMFGELL | TSFVDLTRFLDSGVL | |
| 66 | RLMNNLEFL | HSTSFVDLTRFLDSG | |
| 67 | LSCTFLFMF | LFMFGELLRLMNNLE | |
| 68 | FSVCVLLHSF | LTLFTVLLSCTFLFM | |
| 69 | FPFRRNFEFN | RNFEFNLTHSLLFSV | |
| 70 | FLNHELVKK | VLTLFTVLLSCTFLF | |
| 71 | LNRLFPFRR | RRNFEFNLTHSLLFS | |
| 72 | RLMNNLEFLN | PHSTSFVDLTRFLDS | |
| 73 | FMFGELLRLM | RNFEFNLTHSLLFSV | |
| 74 | GLNRLFPFRR | FNLTHSLLFSVCVLL | |
| 75 | | NFEFNLTHSLLFSVC | |
| 76 | | HSLLFSVCVLLHSFR | |
| 77 | | SLLFSVCVLLHSFRR | |
| 78 | | LLFSVCVLLHSFRRS | |
| 79 | | LFSVCVLLHSFRRSN | |
| 80 | | TVLLSCTFLFMFGEL | |
| 81 | | RRNFEFNLTHSLLFS | |
| 82 | | FMFGELLRLMNNLEF | |
| 83 | | MFGELLRLMNNLEFL | |
| 84 | | FGELLRLMNNLEFLN | |
| 85 | | GELLRLMNNLEFLNH | |
| 86 | | LTHSLLFSVCVLLHS | |
| 87 | | LLSCTFLFMFGELLR | |
| 88 | | VLLSCTFLFMFGELL | |
| 89 | | FRRNFEFNLTHSLLF | |
| 90 | | THSLLFSVCVLLHSF | |

| | | | |
|---|---|---|---|
| 91 | | TRFLDSGVLTLFTVL | |
| 92 | | ELLRLMNNLEFLNHE | |
| 93 | | FEFNLTHSLLFSVCV | |
| 94 | | SFVDLTRFLDSGVLT | |
| 95 | | TSFVDLTRFLDSGVL | |
| 96 | | STSFVDLTRFLDSGV | |
| 97 | | PHSTSFVDLTRFLDS | |
| 98 | | LLRLMNNLEFLNHEL | |
| 99 | | HSTSFVDLTRFLDSG | |
| 100 | | NFEFNLTHSLLFSVC | |
| 101 | | SCTFLFMFGELLRLM | |
| 102 | | FMFGELLRLMNNLEF | |
| 103 | | LLSCTFLFMFGELLR | |
| 104 | | FRRNFEFNLTHSLLF | |
| 105 | | DLTRFLDSGVLTLFT | |
| 106 | | VLLSCTFLFMFGELL | |
| 107 | | LLSCTFLFMFGELLR | |
| 108 | | LSCTFLFMFGELLRL | |
| 109 | | SCTFLFMFGELLRLM | |
| 110 | | CTFLFMFGELLRLMN | |
| 111 | | SFVDLTRFLDSGVLT | |
| 112 | | LTRFLDSGVLTLFTV | |
